# Nuclear quantum effects slow down the energy transfer in biological light-harvesting complexes

**DOI:** 10.1126/sciadv.adw4798

**Published:** 2025-06-06

**Authors:** Johan E. Runeson, David E. Manolopoulos

**Affiliations:** Physical and Theoretical Chemistry Laboratory, Department of Chemistry, University of Oxford, OX1 3QZ Oxford, UK.

## Abstract

We assess how quantum-mechanical effects associated with high-frequency chromophore vibrations influence excitation energy transfer in biological light-harvesting complexes. After defining a classical nuclear limit that is consistent with the quantum-classical equilibrium, we include nuclear quantum effects through a variational polaron transformation of the high-frequency vibrational modes. This approach is validated by comparison with fully quantum-mechanical benchmark calculations and applied to three prototypical light-harvesting complexes. For light-harvesting complex 2 of purple bacteria, the inter-ring transfer is 1.5 times slower in the quantum treatment than in the classical treatment. For the Fenna-Matthews-Olson complex, the transfer rate is the same in both cases, whereas for light-harvesting complex II of spinach, the transfer is 1.7 times slower in the quantum treatment. The effect is most pronounced for systems with large excitonic energy gaps and strong vibronic coupling to high-frequency modes. In all cases, nuclear quantum effects are found to be unimportant for the directionality of energy transfer.

## Teaser

Nuclear quantum effects in light-harvesting complexes slow down rather than increase energy transfer efficiency.

## INTRODUCTION

Photosynthesis is a process of fundamental importance to life on Earth and a source of inspiration for the development of solar fuels. However, there are still some aspects of its mechanism that are open to debate, including the long-standing question of whether biological light harvesting exhibits any quantum effects (*1*–*3*). For almost two decades, this debate focused on the interpretation of oscillatory signals observed in nonlinear electronic spectroscopy experiments (*4*). These signals were originally attributed to inter-exciton coherences, but a consensus has now emerged that they have a more straightforward vibrational or vibronic origin (*5*, *6*). In recent years, the debate has therefore shifted to consider the quantum effects associated with the vibrations themselves (*7*), which we shall refer to here as nuclear quantum effects. Our goal in this article is to contribute to the debate by comparing classical and quantum treatments of the vibrational modes in explicit simulations of the energy transfer in biological light-harvesting complexes.

On the one hand, a series of recent articles have argued that nuclear quantum effects are essential for the function of photosynthetic antenna complexes. For a vibronic model of the light-harvesting complex 2 (LH2) in purple bacteria, Kundu *et al*. (*7*) found that a classical treatment of vibrations provided a poor description of exciton equilibration compared to a fully quantum treatment, which led them to conclude that quantum nuclear motion is necessary for the correct flow of energy. Similar statements have been made by other authors (*8*–*10*), and two recent reviews (*5*, *6*) have identified nuclear quantum effects as responsible for the existence of an energy funnel and therefore for the directionality of energy transfer. On the other hand, numerous simulations with classical nuclei have been shown to be consistent with the exact quantum dynamics of models of the Fenna-Matthews-Olson (FMO) complex of green sulfur bacteria (*11*–*16*), suggesting that nuclear quantum effects do not play any role in its energy transfer. Hence, the literature is still divided on the issue.

To explain why this is, we first need to clarify what we mean by a nuclear quantum effect in the present context. Light-harvesting complexes enable efficient excitation energy transfer through an interplay of dipolar coupling between chromophores and vibronic coupling to nuclear motion. In a fully quantum description, the nuclear degrees of freedom are quantized in the same way as the excitonic degrees of freedom. However, fully quantum dynamics is computationally demanding and only possible for simple model systems, which has motivated the development of approaches that treat the nuclear degrees of freedom classically. An important issue here is that the classical treatment is not unique. Some classical formulations are better justified than others, and different approaches can be more or less accurate in comparison to the quantum description. So only when the best available classical treatment is unable to reproduce the quantum result does it make sense to identify a quantum effect. Such an identification indicates the level of theory that is required—or sufficient—to accurately describe the phenomenon. If one can reproduce the quantum result with a classical simulation, then there is no quantum effect.

The standard quantum description of exciton energy transfer is based on the model Hamiltonian $H=H_{s}+H_{b}+H_{\text{sb}}$ , where $H_{s}$ is the Hamiltonian of the excitonic system, $H_{b}$ is that of a vibrational bath, and $H_{\text{sb}}$ is that of the system-bath interaction. In the basis of locally excited chromophores (or sites), the first term is written as

$$H_{s}=\sum_{n}\varepsilon_{n}|n\rangle \langle n|+\sum_{n\neq m}J_{\mathrm{nm}}|n\rangle \langle m|$$ (1)

where $\varepsilon_{n}$ are the site energies and $J_{\mathrm{nm}}$ are the intersite couplings. The other terms

$$H_{b}=\sum_{\mathrm{nk}}\hbar \omega_{k}\left(b_{\mathrm{nk}}^{†}b_{\mathrm{nk}}+\frac{1}{2}\right)$$ (2)

$$H_{\text{sb}}=\sum_{\mathrm{nk}}\hbar \omega_{k}g_{k}\left(b_{\mathrm{nk}}^{†}+b_{\mathrm{nk}}\right)|n\rangle \langle n|$$ (3)

model how the site energies fluctuate due to interaction with vibrational degrees of freedom with frequencies $\omega_{k}$ and coupling strengths $g_{k}$ . As is customary, we assume that the sites are coupled to identical and independent baths. Typically, one can attribute the high-frequency bath modes to intramolecular vibrations and the low-frequency modes to phonons of the solvent and protein scaffolding.

To identify the role of nuclear quantum effects in this model, one first needs to define an appropriate classical limit for the nuclear motion. On the basis of past literature, it appears that there are many mixed quantum-classical approaches to choose from: surface hopping (*17*), Ehrenfest dynamics, phase-space mappings (*18*, *19*), quantum-classical path integrals (*20*), etc. Each of these methods has its own definition of the force acting on the classical nuclei. The excitonic state-dependent part of this force is the so-called back-action of the quantum system on the classical degrees of freedom. As has been discussed elsewhere (*15*), part of the debate about nuclear quantum effects is semantic in the sense that some authors use the term “classical” to mean “classical with no back-action” or “classical with disregard for Newton’s third law.” It is well known that no back-action leads to equal long-time populations of all quantum states, an unphysical situation that corresponds to elevating the excitonic system to infinite temperature. Neglecting the back-action is therefore inconsistent with the quantum-classical equilibrium distribution at the temperature of interest (*15*).

The fully quantum mechanical Boltzmann population of site *n* is

$$\langle \;|\;n\;\rangle \langle \;n\;|\;\rangle =\frac{{\text{Tr}}_{\text{nuc}}\left[{\text{Tr}}_{\text{ex}}\left[e^{-\beta H}|\;n\;\rangle \;\langle \;n\;|\right]\right]}{{\text{Tr}}_{\text{nuc}}\left[{\text{Tr}}_{\text{ex}}\left[e^{-\beta H}\right]\right]}$$ (4)

where the labels “nuc” and “ex” refer to the nuclear and excitonic degrees of freedom, respectively. The classical analog of the nuclear trace is obtained by replacing $b_{\mathrm{nk}}=\frac{1}{\sqrt{2\hbar \omega_{k}}}\left(\omega_{k}q_{\mathrm{nk}}+{\mathrm{ip}}_{\mathrm{nk}}\right)$ and treating $\left(p,q\right)=\left(\;\{\;p_{\mathrm{nk}}\;\},\{\;q_{\mathrm{nk}}\;\}\;\right)$ as classical variables. The classical limit of the population then emerges from an average of the excitonic Boltzmann distribution over the nuclear phase space

$$\begin{array}{c} {\langle \;|\;n\;\rangle \langle \;n\;|\;\rangle }_{\text{cl}}=\frac{\int \;\text{dpdq}\;{\text{Tr}}_{\text{ex}}\left[e^{-\beta H\left(p,q\right)}|\;n\rangle \langle n\;|\right]}{\int \;\text{dpdq}\;{\text{Tr}}_{\text{ex}}\left[e^{-\beta H\left(p,q\right)}\right]} \end{array}$$ (5)

Although the mixed quantum-classical statistics in Eq. 5 is much simpler than the fully quantum statistics in Eq. 4, very few mixed quantum-classical dynamics methods are actually consistent with it. Ehrenfest dynamics is known to overheat the excitonic subsystem in much the same way as neglecting back-action. Fewest-switches surface hopping often gives more accurate long-time populations than Ehrenfest dynamics, but the stochastic nature of this algorithm makes it hard to analyze the circumstances under which they can be trusted. A recently developed alternative is Mannouch and Richardson’s deterministic “mapping approach to surface hopping” (MASH) (*21*). We have adapted this method to handle multiple electronic states (*22*), successfully applied the adaptation to the energy transfer in FMO (*23*), and proven that it exactly recovers the correct quantum-classical equilibrium populations in Eq. 5 (*22*). Since our multi-state version of MASH is the only method we are aware of that both recovers these equilibrium populations and has been shown to work well for excitonic Hamiltonians, it is the method we shall use for the present calculations.

## RESULTS

Of the many known biological light-harvesting complexes, we have selected three representative examples: LH2 in purple bacteria, FMO in green sulfur bacteria, and the light-harvesting complex II (LHCII) in spinach. All three systems have been studied extensively, both experimentally and theoretically, and the historical debate about nuclear quantum effects has largely been focused on LH2 and FMO. We will consider the physiologically relevant temperature of 300 K in all of our simulations.

### LH2 in purple bacteria

**Fig. 1.**
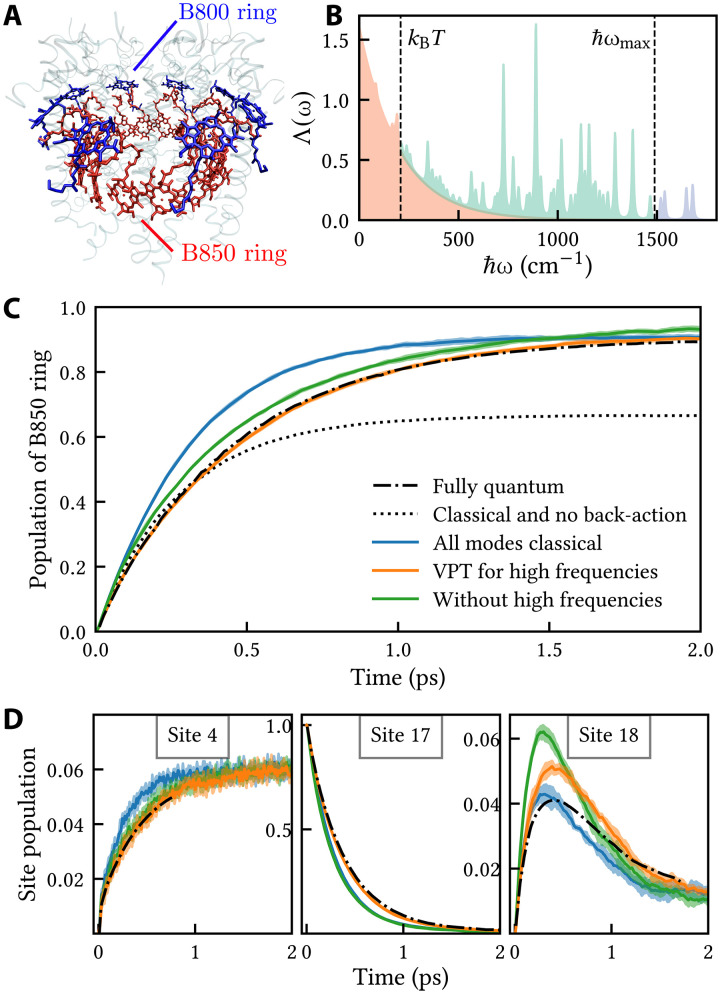
Exciton energy transfer in LH2. (**A**) LH2 structure, composed of the B850 ring (red, sites 1 to 16) and the B800 ring (blue, sites 17 to 24) embedded in a protein scaffolding (shaded). (**B**) Density of bath reorganization energies, $\Lambda \left(\omega \right)=\sum_{k}\hbar \omega_{k}g_{k}^{2}\delta \left(\hbar \omega -\hbar \omega_{k}\right)$ . This dimensionless quantity is partitioned into a low-frequency part that contains the solvent and discrete modes below $k_{B}T$ (red), a high-frequency part containing discrete modes between $k_{B}T$ and $\hbar \omega_{\text{max}}$ (teal), and an off-resonant part containing discrete modes above $\hbar \omega_{\text{max}}$ (purple). For visual purposes, each discrete mode has been broadened by γ = 10 cm^−1^ such that its contribution to Λ(ω) is $\frac{2}{\pi }g_{k}^{2}\omega_{k}^{2}\frac{\gamma \omega }{{\left(\omega_{k}^{2}-\omega^{2}\right)}^{2}+\omega^{2}\gamma^{2}}$ . (**C**) Time-dependent population of the B850 ring after an initial excitation on site 17 in the B800 ring. Shades around lines indicate two standard errors in the mean. (**D**) Time-dependent populations of a few representative sites.

As our first example, we consider LH2 in purple bacteria. This complex consists of two bacteriochlorophyll rings, which have absorption maxima near 800 and 850 nm and are consequently called the B800 and B850 rings. Its structure varies across different species (*24*), and in the following, we focus on the octameric complex found in *Magnetospirillum molischianum* (note that the former generic names *Rhodospirillum* and *Phaeospirillum* are often still used in the literature). In total, the complex comprises 24 chromophores embedded in a protein scaffolding, as depicted in Fig. 1A [using the crystal structure from (*25*)]. The primary function of the complex is to funnel excitation energy inward, from B800 to B850, to reach a reaction center near the center of the B850 ring.

As a fully quantum benchmark for the exciton dynamics, we use the recent path integral calculations by Kundu *et al*. (*7*). Their model Hamiltonian uses site energies and couplings from Tretiak *et al.* (*26*), and a bath spectral density that combines an experimentally determined intramolecular contribution (*27*) with a phenomenological solvent contribution (see note S1 for full details). To illustrate the spectral density, Fig. 1B shows the (dimensionless) density of bath reorganization energies, $\Lambda \left(\omega \right)=\sum_{k}\hbar \omega_{k}g_{k}^{2}\delta \left(\hbar \omega -\hbar \omega_{k}\right)$ . [Note that other common ways to represent the spectral density are through the density of Huang-Rhys factors, $S\left(\omega \right)=\sum_{k}g_{k}^{2}\delta \left(\omega -\omega_{k}\right)$ , and through the spectral function $J\left(\omega \right)=\pi \sum_{k}\hbar \omega_{k}^{2}g_{k}^{2}\delta \left(\omega -\omega_{k}\right)$ , both of which have the same information content as Λ(ω) . We prefer to use Λ(ω) because it is more transparently related to the total bath reorganization energy, $\Lambda =\hbar \int_{0}^{\infty }\Lambda \left(\omega \right)d\omega$.]

After an initial excitation on the B800 ring (assumed to be localized on site 17), the transfer to the B850 ring can be described by rate-like kinetics, as shown in Fig. 1C. In the fully quantum calculation (*7*) (dash-dotted line), the B850 population reaches a long-time value of $P_{\infty }$ = 0.90 with time constant τ=0.45ps , as determined from a fit to $P_{B850}\left(t\right)=P_{\infty }\left(1-e^{-t/\tau }\right)$ . Kundu and colleagues also simulated the dynamics in the limit of classical nuclei, with a method that excludes back-action (shown as the dotted line in Fig. 1C) (*7*, *28*). As we have already mentioned, such a classical approach leads to equal long-time populations on all sites, reducing the long-time yield of the B850 ring to ^16^/_24_ = ^2^/_3_. Since this is less than the quantum yield of 90%, Kundu and colleagues concluded that quantum nuclear motion was responsible for the more efficient energy transfer seen in their path integral calculation (*6*, *7*).

We have included back-action in the classical calculation by running mixed quantum-classical dynamics with MASH. The result of this calculation is shown as the solid blue line in Fig. 1C. MASH is seen to give an accurate long-time B850 yield despite treating the nuclear motion classically. This implies that there is little difference between the fully quantum (Eq. 4) and mixed quantum-classical (Eq. 5) equilibrium distributions for this problem, as can be verified with a simple equilibrium simulation (see note S2). It also shows that it is unreliable to draw conclusions about nuclear quantum effects from comparisons with classical simulations without back-action (*5*, *7*). When back-action is included in the classical simulation, there is no longer any evidence for quantum nuclear motion leading to more efficient energy transfer in the sense of giving a larger B850 yield.

The present calculations do, however, reveal a different nuclear quantum effect. The time constant of the fully quantum calculation in Fig. 1C (τ = 0.45 ps) is almost 50% larger than that of the “all modes classical” MASH calculation (τ = 0.31 ps). Presumably this is because a substantial fraction of the reorganization energy in LH2 comes from vibrational modes with $\hbar \omega >k_{B}T$ (see Fig. 1B), the classical treatment of which neglects their zero point energy. What is less obvious is why switching to a quantum treatment decreases the energy transfer rate. A clue is provided by the similarity between the present excitonic model and the Holstein models that are widely used to describe charge transport in organic semiconductors. In that context, the primary effect of high-frequency on-site modes is to reduce the average intersite couplings (due to vibronic mixing) and thereby reduce the charge mobility (*29*–*31*). For extended systems in which the electronic states form a band, this effect corresponds to a narrowing of the band width. It is therefore natural to ask whether a similar effect is in operation here.

To test this hypothesis, we have used a variational polaron transformation (VPT) (*31*, *32*) (see Methods) to obtain a set of dressed excitonic states that mix bare excitonic states with nuclear modes (the selection of modes will be made precise below). The transformed Hamiltonian is also of system-bath form, $H\prime =H_{s}^{\prime }+H_{b}^{\prime }+H_{\text{sb}}^{\prime }$ , where $H_{s}^{\prime }$ is a modified system Hamiltonian with renormalized site energies ( $\varepsilon_{n}^{\prime }$ ) and couplings ( $J_{\mathrm{nm}}^{\prime }$ ), $H_{b}^{\prime }=H_{b}$ is unchanged, and $H_{\text{sb}}^{\prime }$ contains reduced linear couplings on the diagonal and additional exponential couplings on the off-diagonal. The transformation is variationally optimized to minimize the contribution of $H_{\text{sb}}^{\prime }$ to the free energy (*32*). To account for the remaining bath coupling, other authors have suggested using a perturbative master equation with $H_{\text{sb}}^{\prime }$ as the perturbation (*33*–*37*). However, such an approach is not ideal for the present system for the following reasons. The fluctuations in $H_{\text{sb}}^{\prime }$ are only perturbatively small when the temperature is low (frequencies are high) and the Huang-Rhys factors are sufficiently small. For high temperature (low frequencies) or large Huang-Rhys factors, the renormalized couplings $J_{\mathrm{nm}}^{\prime }$ approach zero and the corresponding fluctuations in $H_{\text{sb}}^{\prime }$ are large (*38*). Low-frequency modes are therefore already better described with MASH [which is nonperturbative and non-Markovian (*23*)] so they should not be included in the VPT. The renormalized system Hamiltonian captures the main effect of the high-frequency modes, and in accordance with common practice for organic semiconductors, we neglect the effect of their remaining fluctuations (which is small compared to the effect of the low-frequency modes).

A natural energy scale to distinguish between “low” and “high” frequencies is $k_{B}T$ . However, it is not ideal to introduce a sharp cutoff in the spectral density, since this corresponds to a bath with unphysically long-lived correlations (*39*). A pragmatic solution is to make use of the existing division between the smooth solvent part and the discrete intramolecular part of the spectral density. In the following, we define the “low-frequency part” to consist of (i) discrete modes with $\hbar \omega_{k}<k_{B}T$ and (ii) the entire solvent part of the spectral density, assuming it is smooth and dominated by frequencies lower than $k_{B}T$ . This is the part of the spectral density that is colored red in Fig. 1B. The “high frequency” part that is included in the VPT is the remainder, which only contains discrete modes with frequencies $\hbar \omega_{k}>k_{B}T$ . This can be further divided into two contributions: the region colored in teal between $k_{B}T$ and $\hbar \omega_{\text{max}}$ , and the region colored in purple beyond $\hbar \omega_{\text{max}}$ , where $\hbar \omega_{\text{max}}$ is the maximum gap between the eigenenergies of $H_{s}$ (1488 cm^−1^ for LH2). The reason for this further subdivision is that the purple part of the spectral density is out of resonance with the exciton dynamics, and so is unlikely to be responsible for any interesting effects. We have confirmed that classical MASH gives the same results when it is run both with and without these nonresonant modes.

The MASH dynamics after the VPT has been applied (orange line in Fig. 1C) is in close agreement with the fully quantum result. This is also the case for the site-specific populations shown in Fig. 1D, with the exception of site 18, which has a turnover where our method overestimates the maximum population. Overall, the VPT has corrected the timescale of the dynamics compared to treating all modes classically. We obtain band-narrowing factors $J_{\mathrm{nm}}^{\prime }/J_{\mathrm{nm}}$ that are on average 0.89 (arithmetic average over sites) and minimally 0.80, corresponding to a mild reduction in the couplings. These band-narrowing factors account for quantum nuclear statistics by virtue of the factor of $\text{coth}\left(\beta \hbar \omega_{k}/2\right)=2n_{k}+1$ in Eq. 16, where $n_{k}={\left(e^{\beta \hbar \omega_{k}}-1\right)}^{-1}$ is the Bose-Einstein distribution (see Methods). Since the only difference between the blue and orange lines in Fig. 1 is whether the high-frequency modes are treated classically (with MASH) or quantum-mechanically (with the VPT), we can identify the change in rate as a nuclear quantum effect. In the classical simulation, the high-frequency modes are actively promoting the energy transfer, whereas in the quantum calculation they are largely frozen in their ground states. Compared to a MASH calculation without the high-frequency modes (the green lines in Fig. 1), treating these modes classically enhances the rate of energy transfer, whereas treating them quantum mechanically inhibits it.

Before continuing to the next system, we should point out that a more popular way to include nuclear quantum effects in system-bath problems is to start the bath variables from a Wigner distribution. When we do this, we find that the resulting MASH dynamics (the gray line in fig. S3) differs from the classical MASH dynamics and is consistent at short times with the slower energy transfer obtained using the VPT. However, since the Wigner distribution effectively initializes the high-frequency modes at an elevated temperature, the resulting long-time populations correspond to an overheated equilibrium. For this reason, using an initial Wigner distribution in MASH is less accurate at long times than combining it with the VPT.

### FMO complex

Our identification of nuclear quantum effects in LH2 stands in contrast to previous studies of another well-studied light-harvesting system, the FMO complex in green sulfur bacteria. For simple FMO models in which the bath is treated as a single overdamped oscillator, many classical trajectory methods [including MASH (*22*) and others (*12*, *13*, *40*)] have been found to give results in good agreement with fully quantum dynamics (*41*). The obvious question, therefore, is why are nuclear quantum effects more important in LH2 than in FMO?

**Fig. 2.**
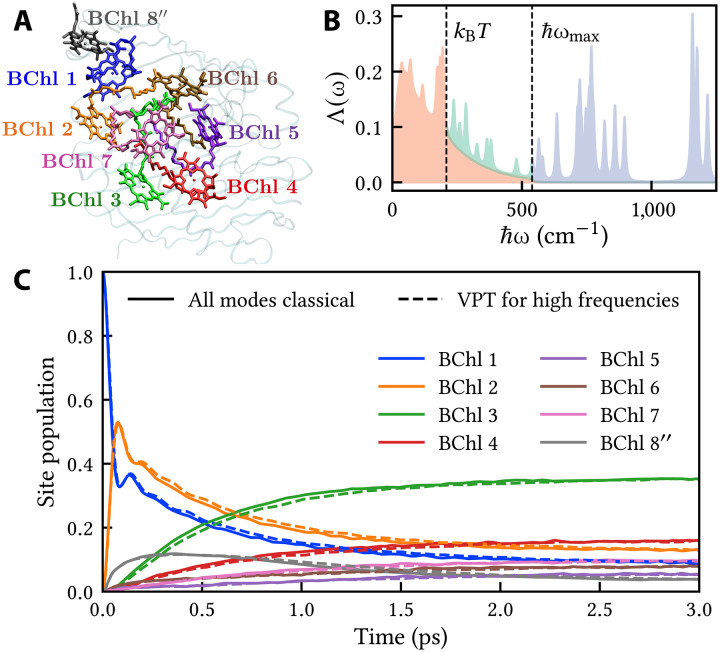
Energy transfer in FMO. (**A**) Site labeling. (**B**) Spectral density plotted as in Fig. 1. (**C**) The MASH population dynamics is essentially the same with or without the VPT.

To answer this question, we consider a standard eight-site model of FMO in *Prosthecochloris aestuarii* with site energies and couplings taken from Schmidt am Busch *et al.* (*42*). The site labeling of the complex is shown in Fig. 2A. The spectral density consists of a continuous intermolecular part (*43*–*45*) and a discrete intramolecular part obtained from fluorescence line-narrowing experiments (*46*). Using the same criteria as for LH2, Fig. 2B shows the division of the spectral density into a low-frequency part (red), a high-frequency part (teal), and a nonresonant part with frequencies larger than $\hbar \omega_{\text{max}}=539\;{\text{cm}}^{-1}$ (purple). Important observations are that (i) the maximum excitonic gap $\hbar \omega_{\text{max}}$ is smaller for FMO than for LH2, primarily because the intersite couplings are smaller, and (ii) the vibronic coupling is weaker in FMO than in LH2 in the region between $k_{B}T$ and $\hbar \omega_{\max}$ . As a result, all the band-narrowing factors $J_{\mathrm{nm}}^{\prime }/J_{\mathrm{nm}}$ are ≥0.94, and the dynamics hardly changes when the high-frequency modes are treated with the VPT instead of classically. To check that the results are insensitive to the details of the spectral density, we have repeated the calculation with a more detailed intermolecular spectrum extracted from a normal-mode analysis of the full atomistic complex (*47*), and reached the same conclusion (see fig. S1). On the basis of these results, we conclude that nuclear quantum effects are negligible in FMO because of its small excitonic energy gaps and its weak vibronic coupling in the relevant “quantum” region between $k_{B}T$ and $\hbar \omega_{\text{max}}$.

### Light-harvesting complex II in spinach

**Fig. 3.**
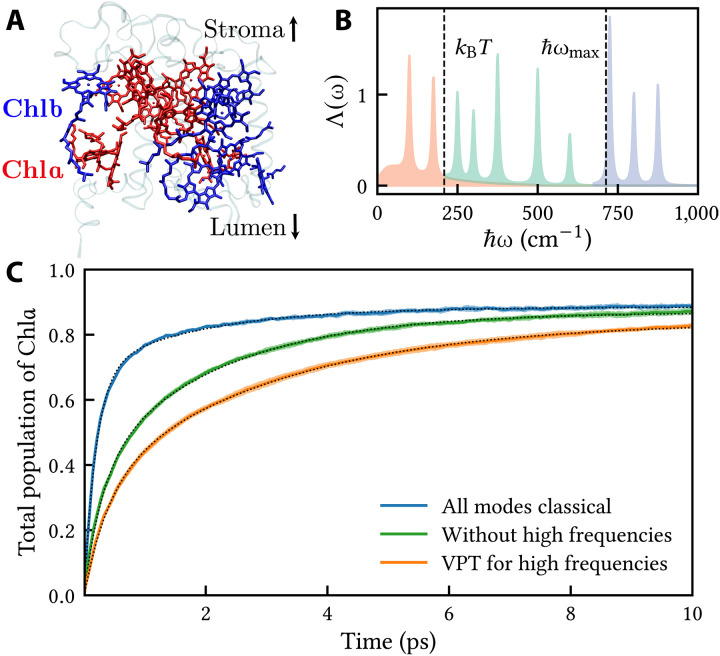
Exciton dynamics in the major LHCII complex of spinach. (**A**) Structure (*49*) showing the assignments of Chla and Chlb chlorophylls in red and blue, respectively, and the orientation relative to the stromal and lumenal layers. (**B**) Spectral density plotted as in Fig. 1. (**C**) Time-dependent population of the Chla sites (dotted lines are biexponential fits).

Having found that nuclear quantum effects are noticeable in LH2 but negligible in FMO, we now ask which of these two pictures is likely to be more representative of photosynthesis in nature? To answer this question, we have chosen to consider the LHCII complex, which is present in more than 50% of all plants (*48*), as our third example. Figure 3A shows the major LHCII complex in spinach (*Spinacia oleracea*) (*49*). It contains 14 chlorophyll sites, which are traditionally divided into two groups, Chla and Chlb. The latter have higher excitation energies, resulting in an energy funnel that is directed toward the Chla sites.

Many different models of this complex have been proposed in the literature. Here, we use the site couplings of Müh *et al.* (*50*), with refined site energies from (*51*) and a spectral density from (*52*) (shown in Fig. 3B). This model reproduces the experimental linear absorption, linear dichroism, and fluorescence spectra. Although there are alternative models that would provide quantum benchmarks (*53*–*55*), we have chosen not to compare with them because their spectral density consists of a single overdamped oscillator dominated by frequencies larger than $k_{B}T$ , which is inconsistent both with our criteria for using the VPT and with the experimental low-temperature fluorescence spectrum (*50*). For the present model, $\hbar \omega_{\text{max}}=713\;{\text{cm}}^{-1}$ is intermediate between the case of LH2 (1488 cm^−1^) and FMO (539 cm^−1^). The same holds for the fraction of reorganization energy in the interval $\left[k_{B}T,\hbar \omega_{\text{max}}\right]$ relative to that in $\left[0,\hbar \omega_{\text{max}}\right]$ , which is 44% for LHCII, 11% for FMO, and 54% for LH2. For the purpose of qualitatively assessing nuclear quantum effects, it is therefore justified to use the same analysis as in the previous examples without reference to another fully quantum benchmark.

For simplicity, we start the simulation from the highest energy site, which is labeled *b*609 in the protein database entry 1RWT (*49*). For each of our methods, the resulting downhill energy transfer to the Chla sites can be fitted to a sum of two exponentials with time constants $\tau_{1}$ and $\tau_{2}$ (the fits are shown with dotted lines in Fig. 3C). When all modes are treated classically with MASH, we obtain $\tau_{1}=0.20$ ps and $\tau_{2}=1.8$ ps. When the high-frequency modes are handled with the VPT, the corresponding results are $\tau_{1}=0.40$ ps and $\tau_{2}=3.0$ ps. Expressed in terms of rates, the associated classical rate constants are 2.0 and 1.7 times larger, respectively, than those obtained with the VPT. In other words, the slowdown associated with nuclear quantum effects is even more pronounced for LHCII than for LH2. As was the case for LH2, a comparison to a calculation without the high-frequency modes (green line) shows that the classical treatment speeds up the transfer, whereas the quantum treatment (VPT) slows it down. This last observation appears at first glance to contradict a previous study (*52*), which found that the discrete part of the spectral density increases the rate. However, if we neglect all discrete modes (gray line), the rate is significantly slower again, so our results do not contradict this previous study, but rather indicate that the speedup is caused mainly by low-frequency discrete modes.

## DISCUSSION

In conclusion, the main difference between quantum and classical treatments of vibrational motion in light-harvesting complexes is not in the direction of energy flow, but instead on the rate of energy transfer. More precisely, it is sufficient to treat the vibrational motion classically to obtain the correct long-time equilibrium, but the rate of transfer will generally be overestimated. Renormalizing the Hamiltonian with the VPT provides a simple way to account for this quantum effect. For biologically relevant parameter regimes, the quantum effect on the rate is benign (less than a factor 2), and the delay may be regarded as a quantum correction to an otherwise qualitatively valid classical picture.

In reaching these conclusions, we have assumed that the coupling to high-frequency vibrations is weak enough to discard the fluctuations of the renormalized VPT Hamiltonian. This assumption is more questionable for processes involving charge transfer (e.g., in the reaction center), but for such processes, the intersite coupling is typically small enough to use Förster theory (the perturbative master equation that results from a full polaron transformation). In comparison to classical Marcus theory, Förster theory can account for both positive and negative quantum corrections to the transfer rates, and the effect can reach orders of magnitude in the inverted regime.

For the present systems, the quantum correction to the energy transfer rate is so slight that the majority of it can be captured simply by neglecting the high-frequency modes rather than treating them classically. In organic semiconductors, the slowdown of the charge mobility due to high-frequency “killer modes” can be much more dramatic (*56*). It is therefore conceivable that chlorophylls have been selected during the evolution of light-harvesting complexes because their excitons are relatively weakly coupled to high-frequency modes.

One of the motivations for studying biological energy transfer is as inspiration for the design of synthetic complexes such as porphyrin nanostructures (*57*–*60*). However, since the porphyrins in these nanostructures are connected by covalent bonds, they may be more strongly affected by high-frequency vibrations than the chlorophylls we have considered here. Our results for biological complexes show that care needs to be taken when treating high-frequency vibrations, and that the VPT provides a simple way to capture their quantum mechanical behavior. We can see no reason why this would not also be the case for synthetic complexes.

## METHODS

### Multi-state MASH

In this mixed quantum-classical method, one starts by replacing the nuclear operators by their classical limit, $b_{\mathrm{nk}}=\frac{1}{\sqrt{2\hbar \omega_{k}}}\left(\omega_{k}q_{\mathrm{nk}}+{\mathrm{ip}}_{\mathrm{nk}}\right)$ , leading to a multi-state Hamiltonian of the (mass-scaled) form

$$H\left(p,q\right)=\frac{p^{2}}{2}+V\left(q\right)$$ (6)

$$V\left(\;q\;\right)=\sum_{n,m}V_{\mathrm{nm}}\left(\;q\;\right)\langle |\;n\;\rangle \langle \;m\;|\;\rangle$$ (7)

The goal is to propagate trajectories of the nuclear variables (p,q) alongside the wavefunction $|\;\psi \;\rangle \;=\;\sum_{n}c_{n}|n\rangle$ , where one may think of the complex coefficients $c_{n}$ as phase-space variables for the excitonic degrees of freedom. Multi-state MASH does this by using the force of the local eigenstate with the largest instantaneous population to propagate the nuclei (*22*). At each timestep, the local eigenstates are found by solving the eigenvalue problem

$$V\left(\;q\;\right)|\;a\left(\;q\;\right)\;\rangle \;=\;V_{a}\left(\;q\;\right)|a\left(\;q\;\right)\;\rangle$$ (8)

and their populations are computed as $P_{a}\;=\;{|\;\langle \;\psi \;|\;a\left(\;q\;\right)\;\rangle \;|}^{2}$ . The force can then be written as

$$\dot{p}\;=\;-\;\sum_{a}\langle \;a\left(\;q\;\right)|\;\nabla \;V\left(\;q\;\right)|a\left(\;q\;\right)\rangle \Theta_{a}\left(\;P\;\right)$$ (9)

where

$$\Theta_{a}\left(P\right)=\left\{\begin{array}{c} 1\;\text{if}\;P_{a}>P_{b}\;\forall b\neq a\\ 0\;\text{otherwise} \end{array}\right.$$ (10)

Only the maximally populated state has a nonzero value of $\Theta_{a}\left(P\right)$ , and we refer to this state as the “active” state. Whenever a new state reaches a higher population than the previously active state, there is an associated jump in the potential energy, and the momentum is adjusted to preserve the total energy. The momentum is rescaled along the particular direction specified in appendix E of (*22*). If the available kinetic energy is insufficient to hop, the momentum is instead reversed along the same direction [analogous to a particle bouncing off a wall (*21*)].

To measure populations, we use the estimator (*22*)

$$\Phi_{n}=\frac{1}{N}+\alpha_{N}\left({|\;c_{n}\;|}^{2}-\frac{1}{N}\right)$$ (11)

where $\alpha_{N}=\left(N-1\right)/\left(H_{N}-1\right)$ and $H_{N}=\sum_{n=1}^{N}\frac{1}{n}$ . This estimator is constructed to be consistent with the mixed quantum-classical equilibrium population in Eq. 5, which the “Ehrenfest population” ${|\;c_{n}\;|}^{2}$ is not. Various initial conditions for the coefficients $c_{n}$ are compatible with this estimator (*23*). In the present calculations, we used the “focused” initial condition in which $c_{n}=\sqrt{r_{n}}e^{i\phi_{n}}$ , where $\phi_{n}$ is sampled uniformly from [0,2π) and $r_{n}$ is chosen so that $\Phi_{n}$ is 1 for the initial state and 0 for all other states. If *i* is the initial state, one obtains $r_{i}=\frac{N+\alpha_{N}-1}{N\alpha_{N}}$ and $r_{n\neq i}=\frac{\alpha_{N}-1}{N\alpha_{N}}$ . The nuclear variables were initialized from a classical Boltzmann distribution centered at *q* = 0, corresponding to a vertical excitation from the ground-state equilibrium. The results were averaged over 10^5^ trajectories for LH2 and LHCII, and 5 × 10^4^ trajectories for FMO. Each simulation was divided into five batches, from which the standard errors in the mean were calculated.

### Variational polaron transformation

The VPT (*32*) is an exact canonical transformation $H\prime =e^{G}{\mathrm{He}}^{-G}$ with the generator

$$G=\sum_{\mathrm{nk}}f_{\mathrm{nk}}\left(b_{\mathrm{nk}}^{†}-b_{\mathrm{nk}}\right)|\;n\;\rangle \;\langle \;n\;|$$ (12)

The transformation leads to another system-bath Hamiltonian $H\prime =H_{s}^{\prime }+H_{b}^{\prime }+H_{\text{sb}}^{\prime }$ , where

$$H_{s}^{\prime }=\sum_{n}\varepsilon_{n}^{\prime }|\;n\;\rangle \langle \;n\;|+\sum_{n\neq m}J_{\mathrm{nm}}^{\prime }|\;n\;\rangle \;\langle \;n\;|$$ (13)

describes an excitonic system with renormalized sit0e0 energies and couplings, $H_{b}^{\prime }=H_{b}$ , and

$$\begin{array}{c} \;H_{\text{sb}}^{\prime }=\sum_{\mathrm{nk}}\hbar \omega_{k}\left(g_{k}-f_{\mathrm{nk}}\right)\left(b_{\mathrm{nk}}^{†}+b_{\mathrm{nk}}\right)|n\rangle \langle n|\\ +\sum_{n\neq m}J_{\mathrm{nm}}\left(B_{\mathrm{nm}}-\langle B_{\mathrm{nm}}\rangle \right)|\;n\;\rangle \;\langle \;m\;| \end{array}$$ (14)

is a renormalized system-bath interaction with $B_{\mathrm{nm}}=e^{+\sum_{\mathrm{nk}}f_{\mathrm{nk}}\left(b_{\mathrm{nk}}^{†}-b_{\mathrm{nk}}\right)-\sum_{\mathrm{mk}}f_{\mathrm{mk}}\left(b_{\mathrm{mk}}^{†}-b_{\mathrm{mk}}\right)}$.

The renormalized site energies and couplings are

$$\varepsilon_{n}^{\prime }=\varepsilon_{n}-\sum_{k}\hbar \omega_{k}\left(2f_{\mathrm{nk}}g_{k}-f_{\mathrm{nk}}^{2}\right)$$ (15a)

$$J_{\mathrm{nm}}^{\prime }=J_{\mathrm{nm}}\langle \;B_{\mathrm{nm}}\;\rangle$$ (15b)

where

$$\langle B_{\mathrm{nm}}\rangle =e^{-\frac{1}{2}\sum_{k}\left(f_{\mathrm{nk}}^{2}+f_{\mathrm{mk}}^{2}\right)\text{coth}\left(\beta \hbar \omega_{k}/2\right)}$$ (16)

is the band narrowing factor.

The aim is to adjust the variational parameters $f_{\mathrm{nk}}$ such that $H_{1}=H_{\text{sb}}^{\prime }$ can be neglected next to $H_{0}\equiv H_{s}^{\prime }+H_{b}^{\prime }$ . According to Bogoliubov’s theorem, the free energy of the total system is bounded from above by the free energy of the uncoupled system, $F\leq F_{0}+{\langle H_{1}\rangle }_{0}$ , where ${\langle H_{1}\rangle }_{0}=0$ by construction for any $f_{\mathrm{nk}}$ . To make $F_{0}$ as close as possible to *F*, one therefore chooses $f_{\mathrm{nk}}$ such that $F_{0}$ is minimized. In practice, this leads to the condition

$$0=\frac{\partial F_{0}}{\partial f_{\mathrm{nk}}}=\sum_{M}P_{M}\frac{\partial \varepsilon_{M}}{\partial f_{\mathrm{nk}}}$$ (17)

where $\varepsilon_{M}=\langle \;M|H_{s}^{\prime }|\;M\;\rangle$ are eigenenergies of $H_{s}^{\prime }$ with eigenstates ∣M〉 , and $P_{M}=e^{-{\beta \varepsilon }_{M}}/\sum_{N}e^{-{\beta \varepsilon }_{N}}$ . Using the Hellmann-Feynman theorem, $\frac{\partial }{\partial \lambda }\langle M|H|M\rangle =\langle M\left|\frac{\partial H}{\partial \lambda }\right|M\rangle$ , one obtains

$$f_{\mathrm{nk}}=\frac{\hbar \omega_{k}g_{k}}{\hbar \omega_{k}-\text{coth}\left(\beta \hbar \omega_{k}/2\right)\sum_{m}^{\prime }J_{\mathrm{nm}}^{\prime }\Gamma_{\mathrm{nm}}}$$ (18)

where $\Gamma_{\mathrm{nm}}=\sum_{M}P_{M}U_{\mathrm{nM}}U_{\mathrm{mM}}/\sum_{M}P_{M}U_{\mathrm{nM}}^{2}$ with $U_{\mathrm{nM}}=\langle \;n\;|\;M\;\rangle$ and the prime on the sum over *m* indicates that terms with m=n are excluded. This expression differs from (*31*) because the exciton states follow a Boltzmann distribution instead of a Fermi-Dirac distribution. Equation 3 was solved self-consistently for $f_{\mathrm{nk}}$ , and a stable solution was found within 10 iterations starting from $f_{\mathrm{nk}}=g_{k}$ . We verified that the solution was insensitive to the choice of initial guess for all systems considered here.

## Supplementary Materials

### This PDF file includes:

Supplementary Text Figs. S1 to S3 References
