# Supplementary material for: Nuclear quantum effects slow down the energy transfer in biological light-harvesting complexes
Source: Sci Adv. 2025 Jun 6;11(23):eadw4798. doi: 10.1126/sciadv.adw4798 (PMC12143380; doi:10.1126/sciadv.adw4798)
Supplement: Supplementary file 1 — Supplementary Text Figs. S1 to S3 References [file sciadv.adw4798_sm.pdf]

Science Advances
AAAS

Supplementary Materials for

Nuclear quantum effects slow down the energy transfer in biological light-harvesting complexes

Johan E. Runeson and David E. Manolopoulos

Corresponding author: Johan E. Runeson, johan.runeson@physik.uni-freiburg.de

Sci. Adv. **11**, eadw4798 (2025)
DOI: 10.1126/sciadv.adw4798

This PDF file includes:

Supplementary Text
Figs. S1 to S3
References

Supplementary Text

Supplementary note 1: Model details

LH2: The input file `LH2-HS.dat` contains the 24×24 matrix of site energies and couplings (*26*) (in cm^{-1}). The file `LH2-specden.dat` contains a table with the discrete part of the spectral density (*27*) with columns $\hbar\omega_k$ (in cm^{-1}) and $g_k^2 * 1000$ (dimensionless), corresponding to a total reorganization energy of 217 cm^{-1}. These files were kindly provided to us by Kundu and Makri. (*7*) The continuous contribution to the spectral density has the phenomenological form (*7*)

$$J(\omega) = \pi \sum_k \hbar\omega_k^2 g_k^2 \delta(\omega - \omega_k) = 2\pi\xi\hbar\omega e^{-\omega/\omega_\mathrm{c}}, \tag{S1}$$

where $\xi = 0.4$ and $\omega_\mathrm{c} = 200\ \mathrm{cm}^{-1}$, which is equivalent to

$$\Lambda(\omega) = \sum_k \hbar\omega_k g_k^2 \delta(\hbar\omega - \hbar\omega_k) = \frac{J(\omega)}{\pi\hbar\omega} = 2\xi e^{-\omega/\omega_\mathrm{c}} \tag{S2}$$

and contributes a total reorganization energy of 160 cm^{-1}. In practice, we discretized the continuous part into $n = 20$ modes with the simple discretization (*61*) $\omega_k = -\omega_\mathrm{c} \log \frac{k+1/2}{n}$, $g_k = \sqrt{\frac{2\xi\omega_\mathrm{c}}{n\omega_k}}$ ($k = 0, \ldots, n-1$). The number n was determined by repeating the calculation for increasingly fine discretization until convergence.

FMO: The input file `FMO-HS.dat` contains the 8×8 matrix of site energies and couplings (*42*). The columns of the file `FMO-specden.dat` contain the values of $\hbar\omega_k$ (in cm^{-1}) and g_k^2 (dimensionless) for the discrete part of the spectral density. (*46*) In the main text, the continuous part of the spectral density was of the form optimized for the B777 complex by Renger and Marcus: (*43, 45*)

$$S(\omega) = \frac{S_0}{s_1 + s_2} \sum_{i=1,2} \frac{s_i}{7!\, 2\omega_i^4} \omega^3 e^{-(\omega/\omega_i)^{1/2}} \tag{S3}$$

where $s_1 = 0.8$, $s_2 = 0.5$, $\hbar\omega_1 = 0.069$ meV, and $\hbar\omega_2 = 0.24$ meV. The overall Huang-Rhys factor was set to $S_0 = 0.42$ for FMO. (*44*) The spectral density of bath reorganization energies is releated to $S(\omega)$ by $\Lambda(\omega) = \omega S(\omega)$. We discretized the continuous bath using an equally spaced grid with $n = 100$ modes up to $\hbar\omega_\mathrm{max} = 540\ \mathrm{cm}^{-1}$, and checked that the results did not change on including more modes. The reorganization energy was 44 cm^{-1} for the discrete part and 38 cm^{-1} for the continuous part of the spectrum.

To check that the results are not sensitive to the particular choice of continuous bath, we have also repeated the calculation replacing the continuous part with a spectral density extracted from a modern normal-mode analysis of the full atomistic complex (*47*). We course-grained the Huang-Rhys factors of the 59367 normal modes into 200 effective modes below $\hbar\omega_{\text{max}} = 540\ \text{cm}^{-1}$. Since Ref. (*47*) reports that the variations of the spectral density between sites are unimportant, we used the site-averaged bath for all sites. The modified spectral density is shown in Fig. S1B. Its reorganization energy, 15 cm^{-1}, is weaker than the bath of the B777 form. For the dynamics (see panel C) one only observes a slight difference between the VPT and classical populations for BChl 3, and the conclusions of the main text are unchanged.

LHCII: The input file `LHCII-HS.dat` contains the couplings from Ref. (*50*) with the refined site energies from Ref. (*51*). To be consistent with previous studies (*50*), we included static disorder in the diagonal terms by sampling site energies for each trajectory from the average in `LHCII-HS.dat` with a standard deviation of 120 cm^{-1}.

The continuous spectral density was of the same form as in Eq. (S3) but with $S_0 = 0.5$, discretized into $n = 200$ modes (*52*). The file `LHCII-specden.dat` contains the discrete spectral density that was constructed as a correction to the continuous form by fitting to the low-frequency fluorescence spectrum (*52*). The upper cut-off $\hbar\omega_{\text{max}}$ was recalculated for each instance of the static disorder.

Supplementary Note 2: Mixed quantum-classical equilibrium

To verify that the mixed quantum-classical equilibrium populations in Eq. (5) provide a close approximation to their quantum mechanical counterparts in Eq. (4) for LH2, we calculated each type of expectation value explicitly as follows. The quantum mechanical thermal expectation value $\langle |n\rangle\langle n| \rangle$ can be calculated exactly from the path-integral expression

$$\langle |n\rangle\langle n| \rangle = \lim_{P\to\infty} \frac{\int d^P q\, e^{-\frac{\beta}{P}\sum_{i=1}^{P}\frac{P^2}{2\hbar^2\beta^2}(q_i-q_{i+1})^2}\text{Tr}_{\text{ex}}\left[\prod_{i=1}^{P} e^{-\frac{\beta}{P}V(q_i)}|n\rangle\langle n|\right]}{\int d^P q\, e^{-\frac{\beta}{P}\sum_{i=1}^{P}\frac{P^2}{2\hbar^2\beta^2}(q_i-q_{i+1})^2}\text{Tr}_{\text{ex}}\left[\prod_{i=1}^{P} e^{-\frac{\beta}{P}V(q_i)}\right]} \tag{S4}$$

where P is the number of imaginary time ring polymer beads. The corresponding mixed quantum-classical expectation value is obtained by replacing $P = 1$ in this expression. We calculated each

quantity with Monte Carlo using 10^5 samples (divided into 10 batches to calculate standard errors of the mean). The quantum expectation values were calculated with $P = 8$, which agreed closely with $P = 4$. The results are shown for each site in Fig. S2. Also shown for comparison are the expectation values for the 'bare' excitonic system described by H_s without any coupling to the bath. As has been shown previously (*62*), the bath does notably change the equilibrium populations compared to the bare excitonic system. However, the difference between treating the bath with quantum or classical statistics is negligible. For this reason, mixed quantum-classical methods such as MASH that are consistent with Eq. (5) are bound to agree with an exact quantum mechanical calculation of the chromophore populations at long times (assuming that the mixed quantum-classical dynamics is sufficiently ergodic to reach thermal equilibrium, as it invariably is in these excitonic systems).

Supplementary Note 3: Wigner distribution

Starting from a thermal Wigner distribution effectively initializes each bath mode k at a different elevated temperature corresponding to

$$\beta_k = \beta \frac{\tanh(\beta\omega_k/2)}{\beta\omega_k/2}. \tag{S5}$$

The exciton dynamics of LH2 obtained using this initialization is compared to the result obtained starting from a classical Boltzmann distribution (as was done in the main text) in Fig. S3.

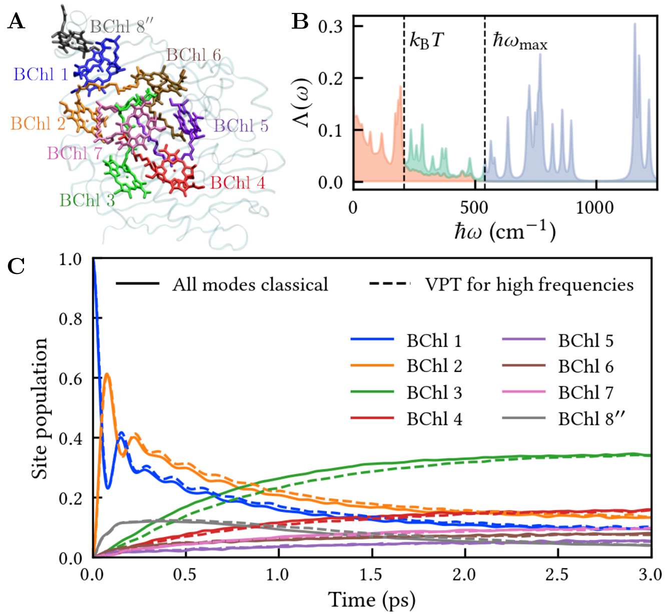

Figure S1: **FMO with a more detailed low-frequency part of the spectral density.** (**A**) Site labelling. (**B**) Modified spectral density. (**C**) Again, the polaron transformation does not notably change the MASH population dynamics.

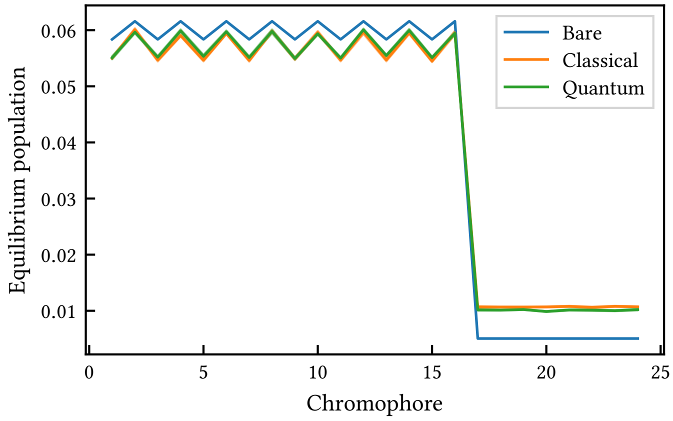

Figure S2: Equilibrium populations of LH2 chromphores computed with classical (P = 1) and quantum (P = 8) nuclei as described in Supplementary Note 2. Shaded areas denote two standard errors in the mean. Also shown for comparison are the bare populations without any coupling to the vibrational bath.

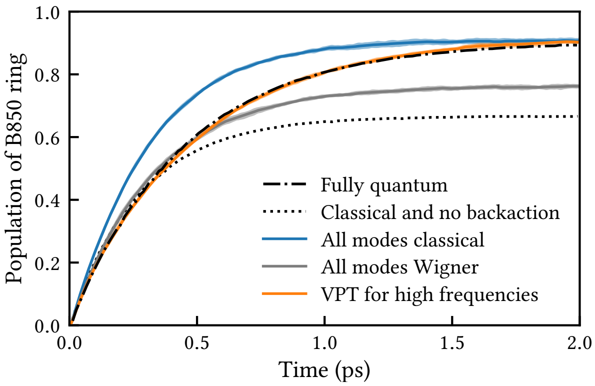

Figure S3: Same as in Figure 1C of the text, but including MASH dynamics starting from a Wigner distribution (gray line). The MASH dynamics starting from a classical distribution reaches the correct mixed quantum-classical equilibrium, which provides a close approximation to the exact quantum mechanical equilibrium for the reasons explained in Supplementary Note 2. However, the long-time dynamics obtained from the Wigner initial conditions reaches an overheated equilibrium, in much the same way as a classical calculation without any back-action (although for different reasons). This problem is solved by treating the high-frequency modes with the VPT and the remainder with classical MASH, which fixes both the long-time equilibrium and the timescale of the relaxation.

REFERENCES AND NOTES

1. N. Lambert, Y.-N. Chen, Y.-C. Cheng, C.-M. Li, G.-Y. Chen, F. Nori, Quantum biology. *Nat. Phys.* **9**, 10–18 (2013).

2. J. S. Higgins, L. T. Lloyd, S. H. Sohail, M. A. Allodi, J. P. Otto, R. G. Saer, R. E. Wood, S. C. Massey, P.-C. Ting, R. E. Blankenship, G. S. Engel, Photosynthesis tunes quantum-mechanical mixing of electronic and vibrational states to steer exciton energy transfer. *Proc. Natl. Acad. Sci. U.S.A.* **118**, e2018240118 (2021).

3. Y. Kim, F. Bertagna, E. M. D'Souza, D. J. Heyes, L. O. Johannissen, E. T. Nery, A. Pantelias, A. Sanchez-Pedreño Jimenez, L. Slocombe, M. G. Spencer, J. Al-Khalili, G. S. Engel, S. Hay, S. M. Hingley-Wilson, K. Jeevaratnam, A. R. Jones, D. R. Kattnig, R. Lewis, M. Sacchi, N. S. Scrutton, S. R. P. Silva, J. McFadden, Quantum biology: An update and perspective. *Quantum Rep.* **3**, 80–126 (2021).

4. G. S. Engel, T. R. Calhoun, E. L. Read, T.-K. Ahn, T. Mančal, Y.-C. Cheng, R. E. Blankenship, G. R. Fleming, Evidence for wavelike energy transfer through quantum coherence in photosynthetic systems. *Nature* **446**, 782–786 (2007).

5. J. Cao, R. J. Cogdell, D. F. Coker, H.-G. Duan, J. Hauer, U. Kleinekathöfer, T. L. C. Jansen, T. Mančal, R. J. D. Miller, J. P. Ogilvie, V. I. Prokhorenko, T. Renger, H.-S. Tan, R. Tempelaar, M. Thorwart, E. Thyrhaug, S. Westenhoff, D. Zigmantas, Quantum biology revisited. *Sci. Adv.* **6**, eaaz4888 (2020).

6. J. D. Schultz, J. L. Yuly, E. A. Arsenault, K. Parker, S. N. Chowdhury, R. Dani, S. Kundu, H. Nuomin, Z. Zhang, J. Valdiviezo, P. Zhang, K. Orcutt, S. J. Jang, G. R. Fleming, N. Makri, J. P. Ogilvie, M. J. Therien, M. R. Wasielewski, D. N. Beratan, Coherence in chemistry: Foundations and frontiers. *Chem. Rev.* **124**, 11641–11766 (2024).

7. S. Kundu, R. Dani, N. Makri, Tight inner ring architecture and quantum motion of nuclei enable efficient energy transfer in bacterial light harvesting. *Sci. Adv.* **8**, eadd0023 (2022).

8. T. Mančal, Excitation energy transfer in a classical analogue of photosynthetic antennae. *J. Phys. Chem. B* **117**, 11282–11291 (2013).

9. M. Reppert, P. Brumer, Quantumness in light harvesting is determined by vibrational dynamics. *J. Chem. Phys.* **149**, 234102 (2018).

10. T. Renger, Semiclassical modified redfield and generalized Förster theories of exciton relaxation/transfer in light-harvesting complexes: The quest for the principle of detailed balance. *J. Phys. Chem. B* **125**, 6406–6416 (2021).

11. G. Tao, W. H. Miller, Semiclassical description of electronic excitation population transfer in a model photosynthetic system. *J. Phys. Chem. Lett.* **1**, 891–894 (2010).

12. M. K. Lee, P. Huo, D. F. Coker, Semiclassical path integral dynamics: Photosynthetic energy transfer with realistic environment interactions. *Annu. Rev. Phys. Chem.* **67**, 639–668 (2016).

13. S. J. Cotton, W. H. Miller, The symmetrical quasi-classical model for electronically non-adiabatic processes applied to energy transfer dynamics in site-exciton models of light-harvesting complexes. *J. Chem. Theory Comput.* **12**, 983–991 (2016).

14. M. A. C. Saller, A. Kelly, J. O. Richardson, Improved population operators for multi-state nonadiabatic dynamics with the mixed quantum-classical mapping approach. *Faraday Discuss.* **221**, 150–167 (2020).

15. J. E. Runeson, J. E. Lawrence, J. R. Mannouch, J. O. Richardson, Explaining the efficiency of photosynthesis: Quantum uncertainty or classical vibrations? *J. Phys. Chem. Lett.* **13**, 3392–3399 (2022).

16. J. E. Lawrence, J. R. Mannouch, J. O. Richardson, A size-consistent multi-state mapping approach to surface hopping. *J. Chem. Phys.* **160**, 244112 (2024).

17. J. C. Tully, Molecular dynamics with electronic transitions. *J. Chem. Phys.* **93**, 1061–1071 (1990).

18. G. Stock, M. Thoss, Classical description of nonadiabatic quantum dynamics. *Adv. Chem. Phys.* **131**, 243–376 (2005).

19. W. H. Miller, S. J. Cotton, Classical molecular dynamics simulation of electronically non-adiabatic processes. *Faraday Discuss.* **195**, 9–30 (2016).

20. R. Lambert, N. Makri, Quantum-classical path integral. I. Classical memory and weak quantum nonlocality. *J. Chem. Phys.* **137**, 22A552 (2012).

21. J. R. Mannouch, J. O. Richardson, A mapping approach to surface hopping. *J. Chem. Phys.* **158**, 104111 (2023).

22. J. E.Runeson, D. E.Manolopoulos, A multi-state mapping approach to surface hopping. *J. Chem. Phys.* **159**, 094115 (2023).

23. J. E. Runeson, T. P. Fay, D. E. Manolopoulos, Exciton dynamics from the mapping approach to surface hopping: Comparison with Förster and Redfield theories. *Phys. Chem. Chem. Phys.* **26**, 4929–4938 (2024).

24. A. L. Tong, O. C. Fiebig, M. Nairat, D. Harris, M. Giansily, A. Chenu, J. N. Sturgis, G. S. Schlau-Cohen, Comparison of the energy-transfer rates in structural and spectral variants of the B800-850 complex from purple bacteria. *J. Phys. Chem. B* **124**, 1460–1469 (2020).

25. J. Koepke, X. Hu, C. Muenke, K. Schulten, H. Michel, The crystal structure of the light-harvesting complex II (B800-850) from *Rhodospirillum molischianum*. *Structure* **4**, 581–597 (1996).

26. S. Tretiak, C. Middleton, V. Chernyak, S. Mukamel, Bacteriochlorophyll and carotenoid excitonic couplings in the LH2 system of purple bacteria. *J. Phys. Chem. B* **104**, 9540–9553 (2000).

27. M. Rätsep, Z.-L. Cai, J. R. Reimers, A. Freiberg, Demonstration and interpretation of significant asymmetry in the low-resolution and high-resolution Qy fluorescence and absorption spectra of bacteriochlorophyll a. *J. Chem. Phys.* **134**, 024506 (2011).

28. N. Makri, Quantum-classical path integral: A rigorous approach to condensed phase dynamics. *Int. J. Quantum Chem.* **115**, 1209–1214 (2015).

29. T. Nematiaram, A. Troisi, Modeling charge transport in high-mobility molecular semiconductors: Balancing electronic structure and quantum dynamics methods with the help of experiments. *J. Chem. Phys.* **152**, 190902 (2020).

30. J. H. Fetherolf, D. Golež, T. C. Berkelbach, A unification of the holstein polaron and dynamic disorder pictures of charge transport in organic crystals. *Phys. Rev. X* **10**, 021062 (2020).

31. Y.-C. Wang, Y. Zhao, Variational polaron transformation approach toward the calculation of thermopower in organic crystals. *Phys. Rev. B* **101**, 075205 (2020).

32. D. Yarkony, R. Silbey, Comments on exciton phonon coupling: Temperature dependence. *J. Chem. Phys.* **65**, 1042–1052 (1976).

33. S. Jang, Y.-C. Cheng, D. R. Reichman, J. D. Eaves, Theory of coherent resonance energy transfer *J. Chem. Phys.* **129**, 101104 (2008).

34. D. P. S. McCutcheon, A. Nazir, Consistent treatment of coherent and incoherent energy transfer dynamics using a variational master equation. *J. Chem. Phys.* **135**, 114501 (2011).

35. E. N. Zimanyi, R. J. Silbey, Theoretical description of quantum effects in multi-chromophoric aggregates. *Philos. Trans. A. Math. Phys. Eng. Sci.* **370**, 3620–3637 (2012).

36. A. Kimura, Y. Fujihashi, Quantitative correction of the rate constant in the improved variational master equation for excitation energy transfer. *J. Chem. Phys.* **141**, 194110 (2014).

37. C. K. Lee, J. Moix, J. Cao, Coherent quantum transport in disordered systems: A unified polaron treatment of hopping and band-like transport *J. Chem. Phys.* **142**, 164103 (2015).

38. A. Troisi, Quantum dynamic localization in the Holstein Hamiltonian at finite temperatures. *Phys. Rev. B* **82**, 245202 (2010).

39. T. C. Berkelbach, D. R. Reichman, T. E. Markland, Reduced density matrix hybrid approach: An efficient and accurate method for adiabatic and non-adiabatic quantum dynamics. *J. Chem. Phys.* **136**, 034113 (2012).

40. J. E. Runeson, J. O. Richardson, Generalized spin mapping for quantum-classical dynamics. *J. Chem. Phys.* **152**, 084110 (2020).

41. A. Ishizaki, G. R. Fleming, Theoretical examination of quantum coherence in a photosynthetic system at physiological temperature. *Proc. Nat. Acad. Sci. U.S.A.* **106**, 17255–17260 (2009).

42. M. Schmidt am Busch, F. Müh, M. El-Amine Madjet, T. Renger, The eighth bacteriochlorophyll completes the excitation energy funnel in the FMO protein. *J. Phys. Chem. Lett.* **2**, 93–98 (2011).

43. T. Renger, R. A. Marcus, On the relation of protein dynamics and exciton relaxation in pigment-protein complexes: An estimation of the spectral density and a theory for the calculation of optical spectra. *J. Chem. Phys.* **116**, 9997–10019 (2002).

44. J. Adolphs, T. Renger, How proteins trigger excitation energy transfer in the FMO complex of green sulfur bacteria. *Biophys. J.* **91**, 2778–2797 (2006).

45. A. Klinger, D. Lindorfer, F. Müh, T. Renger, Living on the edge: Light-harvesting efficiency and photoprotection in the core of green sulfur bacteria. *Phys. Chem. Chem. Phys.* **25**, 18698–18710 (2023).

46. M. Wendling, T. Pullerits, M. A. Przyjalgowski, S. I. E. Vulto, T. J. Aartsma, R. van Grondelle, H. van Amerongen, Electron−Vibrational coupling in the Fenna−Matthews−Olson complex of *Prosthecochloris aestuarii* determined by temperature-dependent absorption and fluorescence line-narrowing measurements. *J. Phys. Chem. B* **104**, 5825–5831 (2000).

47. A. Klinger, D. Lindorfer, F. Müh, T. Renger, Normal mode analysis of spectral density of FMO trimers: Intra- and intermonomer energy transfer. *J. Chem. Phys.* **153**, 215103 (2020).

48. R. E. Blankenship, *Molecular mechanisms of photosynthesis* (Blackwell Science Oxford, 2002).

49. Z. Liu, H. Yan, K. Wang, T. Kuang, J. Zhang, L. Gui, X. An, W. Chang, Crystal structure of spinach major light-harvesting complex at 2.72 Å resolution. *Nature* **428**, 287–292 (2004).

50. F. Müh, M. E.-A. Madjet, T. Renger, Structure-based identification of energy sinks in plant light-harvesting complex II. *J. Phys. Chem. B* **114**, 13517–13535 (2010).

51. F. Müh, T. Renger, Refined structure-based simulation of plant light-harvesting complex II: Linear optical spectra of trimers and aggregates. *Biochim. Biophys. Acta* **1817**, 1446–1460 (2012).

52. T. Renger, M. Madjet, A. Knorr, F. Müh, How the molecular structure determines the flow of excitation energy in plant light-harvesting complex II. *J. Plant Physiol.* **168**, 1497–1509 (2011).

53. C. Kreisbeck, T. Kramer, A. Aspuru-Guzik, Scalable high-performance algorithm for the simulation of exciton dynamics. Application to the light-harvesting complex II in the presence of resonant vibrational modes. *J. Chem. Theory Comput.* **10**, 4045–4054 (2014).

54. X. Leng, Y.-M. Yan, R.-D. Zhu, K. Song, Y.-X. Weng, Q. Shi, Simulation of the two-dimensional electronic spectroscopy and energy transfer dynamics of light-harvesting complex II at ambient temperature. *J. Phys. Chem. B* **122**, 4642–4652 (2018).

55. V. I. Novoderezhkin, Excitation energy equilibration in a trimeric LHCII complex involves unusual pathways. *Phys. Chem. Chem. Phys.* **25**, 26360–26369 (2023).

56. M. A. Dettmann, L. S. R. Cavalcante, C. A. Magdaleno, A. J. Moulé, Catching the killer: Dynamic disorder design rules for small-molecule organic semiconductors. *Adv. Funct. Mater.* **33**, 2213370 (2023).

57. C.-K. Yong, P. Parkinson, D. V. Kondratuk, W.-H. Chen, A. Stannard, A. Summerfield, J. K. Sprafke, M. C. O'Sullivan, P. H. Beton, H. L. Anderson, L. M. Herz, Ultrafast delocalization of excitation in synthetic light-harvesting nanorings. *Chem. Sci.* **6**, 181–189 (2015).

58. A. Pannwitz, O. S. Wenger, Proton-coupled multi-electron transfer and its relevance for artificial photosynthesis and photoredox catalysis. *Chem. Commun.* **55**, 4004–4014 (2019).

59. J. Pruchyathamkorn, W. J. Kendrick, A. T. Frawley, A. Mattioni, F. Caycedo-Soler, S. F. Huelga, M. B. Plenio, H. L. Anderson, A complex comprising a cyanine dye rotaxane and a porphyrin nanoring as a model light-harvesting system. *Angew. Chem. Int. Ed. Engl.* **59**, 16455–16458 (2020).

60. T. Keijer, T. Bouwens, J. Hessels, J. N. H. Reek, Supramolecular strategies in artificial photosynthesis. *Chem. Sci.* **12**, 50–70 (2021).

61. I. R. Craig, D. E. Manolopoulos, Chemical reaction rates from ring polymer molecular dynamics. *J. Chem. Phys.* **122**, 084106 (2005).

62. J. M. Moix, Y. Zhao, J. Cao, Equilibrium-reduced density matrix formulation: Influence of noise, disorder, and temperature on localization in excitonic systems. *Phys. Rev. B* **85**, 115412 (2012).
